# Does frequent hemodialysis regimen results in regression of left ventricular mass compared to conventional hemodialysis?

**Published:** 2015-01-01

**Authors:** Chaudhary Muhammad Junaid Nazar, Faisal Bashir, Syed Ahtizaz Ahmed, Saba Izhar

**Affiliations:** ^1^Department of Endocrinology, University of Buckingham, Royal Gwent Hospital, NHS Trust, Wales, UK; ^2^Department of ENT, New City Teaching Hospital, Mohetarma Benazir Bhutto Shaheed Medical College, Mirpur Azad Kashmir, Pakistan; ^3^Department of Internal Medicine, Allma Iqbal Memorial Teaching Hospital Sialkot, Punjab, Pakistan; ^4^Department of Community Medicine, Nawaz Sharif Medical College, Gujarat, Pakistan

**Keywords:** Hemodialysis, Cardiovascular disease, Chronic kidney disease, Left ventricular mass

## Abstract

Several advances in dialysis therapies have been made. Still, the mortality in end-stage renal disease (ESRD) remains high at rates exceeding 15%. Cardiovascular disease from heart failure or sudden death remains an important cause for mortality in these groups. The most common cardiac anomaly in ESRD is cardiac hypertrophy and this has been observed in 75% of patients at the time of starting dialysis. Also, in patients on conventional hemodialysis (CHD) (4 hours, 3 times per week), left ventricular hypertrophy (LVH) is an independent risk factor for mortality, arrhythmias, heart failure and myocardial ischemia. Stroke work index and left ventricular mass (LVM) are closely associated, in ESRD.

**Implication for health policy/practice/research/medical education:**

Several advances in dialysis therapies have been made. Still, the mortality in end-stage renal disease (ESRD) remains high at rates exceeding 15%. Cardiovascular disease from heart failure or sudden death remains an important cause for mortality in these groups. The most common cardiac anomaly in ESRD is cardiac hypertrophy and this has been observed in 75% of patients at the time of starting dialysis.

## Introduction

Several advances in dialysis therapies have been made. Still, the mortality in end-stage renal disease (ESRD) remains high at rates exceeding 15% (1). Cardiovascular disease from heart failure or sudden death remains an important cause for mortality in these groups (1). The most common cardiac anomaly in ESRD is cardiac hypertrophy and this has been observed in 75% of patients at the time of starting dialysis (2). Also, in patients on conventional hemodialysis (CHD) (4 hours, 3 times per week), left ventricular hypertrophy (LVH) is an independent risk factor for mortality, arrhythmias, heart failure and myocardial ischemia (3). Stroke work index and left ventricular mass (LVM) are closely associated, in ESRD (4). Early work by Sibelberg *et al.* (5) suggested that decreasing the LVM may result in improved survival. The aetiology for LVH in ESRD is multifactorial, contributing factors being anemia, hypertension, volume overload, sleep apnea and uremia. The HEMO study (6) established that increasing the dialysis dose, made no difference to mortality in this group of patients receiving conventional hemodialysis. What about increasing the time or the frequency? This is the subject of the literature review. LVM is a useful surrogate marker for mortality (6) and cardiac magnetic resonance (MR) is a more reliable tool for measuring ventricular mass (5), as observer variability is diminished compared with echocardiography as a diagnostic/study tool.

## Aim/Research question

The aim of the paper work is to review the literature on, impact of frequent hemodialysis on LVM and develop a proposal for a quantitative study that examines the effect of frequent hemodialysis (HD) on regression of LVH compared with CHD regimen, and its association with other physiological parameters namely, blood pressure (BP), extracellular fluid (ECF) volume, anemia and mineral metabolism.

## Literature review

Several medical literature sources have been reviewed, including electronic healthcare databases, Google Scholar. To establish the background, references of references were also reviewed.

The literature available is studded with numerous observational studies on outcomes from frequent dialysis programs, both daily and nocturnal. Back in the 1980s, the association of LVH (7) with mortality was reported and since then, there has been a proliferation of case-control and cohort studies looking into this aspect and its association with other physiological parameters. These observations have reported several advantages, but barring one recent randomized controlled trial (RCT) (1); daily or nocturnal hemodialysis (DHD, NHD) has not been effectively compared to CHD in RCTs.

Since 1998, frequent HD programs earned great attention worldwide. Typically, in most of the observational studies, a DHD regimen would be 2 hours/session, 6 days/week, a NHD regimen 6-8 hours/session, 5-6 nights/week and CHD regimen is 3-4 hours/session, 3 days/week. The treatment is offered in-center, at home or in a self-care center.

Though several studies have reported advantages, the findings are variable. Initially, studies were conducted to look at survival cofactors in small groups of patients. These survival cofactors include systolic/diastolic BPs (both pre and post dialysis), left ventricular mass index (LVMI), parathyroid hormone, hemoglobin, cholesterol, predialysis serum creatinine (8). Subsequently, soft and intermediate end points of LVMI regression, extracellular volume, and anemia were introduced in a few studies (9,10).

Culleton et al. (11) reported from a randomized two period crossover study that involved 12 patients who had been stable on CHD for 6 months or more that DHD allowed better BP control, reduction in LVMI and reduction in antihypertensive therapy, all relating to better ECF control.

DuBois et al. (12)reported from their observation of a cohort of 28 patients switched from CHD to NHD for 2 years at least and a control of 13 patients on CHD for 1 year or more. Regression of LVH, better hemoglobin, BP was reported with no changes in ECF volume. Frequent NHD resulted in reduction in LVMI relating to better systolic blood pressure (SBP) (p= 0.001).

Guyatt et al. (13) reported their 6 years’ experience with 17 patients on short DHD and found that early improvements of amelioration of LVH persist in the mid and long term.

Likewise, Myerson et al. (5) reported yet another non-randomized, controlled trial where 26 patients receiving short DHD were compared with 51 matched conventional HD patients and a 30% decrease in LVMI was found, together with statistically significant reductions in mean C-reactive protein levels. The underlying theme from the cohort studies was clear-there is advantages in frequent hemodialysis in reducing LVMI. Other physiological parameters changed variably.

However, common to all of these studies was methodological flaw. As noted above, they are all observational in nature. The sample sizes were invariably small. The control groups were not necessarily similar and inherently had selection bias (10). Studies did not include patients who may most benefit from frequent hemodialysis; the ill, malnourished, diabetics, those who suffered consequences of intra-dialysis hypotension, the aged (>75) and not necessarily the ‘stable’ ones who could manage long hemodialysis at home. Echocardiography has been employed to assess the LVMI, this is bound to suffer from inter-observer variability (10) and the technique is taken over by cardiac MR studies (5). The measurement of physiological parameters of ECF volume, BP, KT/V, PTH etc. have been inconsistent due to varied definitions and some such as KT/V has not been validated yet in DHD. Statistical analyzer was not blinded to the outcomes from the trials.

One RCT on the subject has been done and has been reviewed in detail. Culleton et al. (11) published the reports of the first RCT in JAMA, 2007. They studied the effect of frequent NHD *versus* CHD on LVM (primary outcome) and quality of life, BP, mineral metabolism and use of antihypertensive (secondary outcome). They concluded that NHD improved LVM, reduced BP medication, improved some measures of mineral metabolism and improved some selected measures of quality of life. 52 patients were chosen who were eligible. Eligibility included adults who had the physical and mental capability to train for nocturnal hemodialysis. This begs a question as to the details of physical capacity-medical conditions, access, and training. The groups were randomized using computer generated sequence.

Dialysis prescriptions, treatment of hypertension on the basis of algorithm, anemia and mineral metabolism parameters and definitions have been clearly stated. However, time to measurement of BP post-dialysis has not been mentioned, and this can be variable. Cardiac MR was employed to assess LV mass, using a standardized formula (11). The reported reduction in LVM was statistically significant p<0.05, although the confidence intervals were relatively wide (-29.6 to -1.0). A significant factor in HD patients that has a bearing on LVMI is the ECF volume. This was not measured at all. The key problem with this study is the small sample size which was not adequately powered to answer the questions relating to quality of life; a secondary outcome measure and adverse events rates. Also, the duration of follow up of these patients was only 6 months, which is again a short period to establish any serious events/safety or other major cardiovascular outcomes.

Hence, there is a need to answer the research question in a RCT that is able to address the deficiencies stated above in the previous RCT with a view to bringing a change in the policy and practice of hemodialysis.

## Methodology

There are generally, two-research paradigms – qualitative and quantitative. Quantitative research, begins with a hypothesis and then, through measurement, generates data, and by deduction, allows a conclusion to be drawn (10). Qualitative research begins with an intention to explore a particular area, collects data and generates ideas and hypothesis from these data through inductive reasoning. The strength of the quantitative research lies in its reliability (repeatability) and that of qualitative research in its validity (closeness to truth) (10). Greenhalgh et al. have disseminated information on incorporating qualitative research into health care. More recently many authors have argued for a combined approach (9,10). Interesting new ideas may be generated which may be then tested with subsequent quantitative research.

For the purpose of the proposed study, a quantitative approach will be undertaken.

## Study design

The study design appropriate to answer our research question will be a rigorous quantitative study design - a RCT with parallel study groups (10), as several small observational studies have already been done with inconsistent findings. The study will seek to falsify or establish the hypothesis that frequent HD causes regression of LVM and look into variables associated with LVMI. It is un-blinded to the researchers and participants, but the data analyzer/radiographer will be blinded. It will also in the process report the safety, efficacy and adverse event rates associated with the practice of frequent HD. An RCT is important in this field as outcomes may influence major policy changes in the way hemodialysis is administered.

## Ethical approval

There is now a quality and accountability framework within which research is to be undertaken in the NHS. This framework is described in the department of health and research governance framework for health and social care. The decision that a research project may proceed is an important management responsibility involving the availability of resources, financial implications, and ethical issues. Before undertaking or hosting any research, an NHS organization must ensure that a favorable opinion on the ethics of the proposed research has been obtained from an appropriate REC (10-14).

Therefore, LREC/MREC committee would be approached for approval, as this study is likely to be multicenter, to be adequately powered. Fully informed consent will be obtained from eligible patients. This is especially important as compliance to the study protocol is important to outcomes. Considering that the outcomes may be policy changing, the multi research ethics committee (MREC) approval is likely to give approval. However, the drawbacks are that, this is a resource intensive trial and is likely to be expensive.

## Study participants

The study population chosen for any trial should reflect the population for whom the therapy is intended (13). Therefore, the general hemodialysis population would tend to include, the seriously ill who may benefit from better clearances, those who suffer from frequent episodes of intra-dialysis hypotension, and patients with recurring pulmonary edema. These patients may benefit from daily in center dialysis. To be able to successfully carry out frequent dialysis at home, the relatively younger population, stable and motivated patients tend to be preferred. This is also suggested by previous studies on frequent hemodialysis (14,15).

So, the study should include patients from wider age groups and some co-morbidities should not preclude the offer of frequent hemodialysis to them. In this way, we can assume that our results would apply to most patients in the general hemodialysis population who could theoretically be included in our study group (16).

## Sample size

Sample size must be planned carefully to ensure that the research time, patient effort and support costs invested in any clinical trial are not wasted (17). Ideally, clinical trials should be large enough to detect reliably the smallest possible differences in the primary outcome with treatment that are considered clinically worthwhile. The minimum information needed to calculate sample size for a RCT in which a specific event is being counted includes the power, the level of significance, the underlying event rate in the population under investigation and the size of the treatment effect sought. The calculated sample size should then be adjusted for other factors, including expected compliance rates and, less commonly, an unequal allocation ratio (18). In order to evaluate the outcomes of frequent hemodialysis, hard end points such as mortality requires a very large sample size and long follow up. Here, financial implications on the funding body, as well as safety issues need to be considered. Therefore, surrogate end point such as LVM regression is a suitable alternative (19). The influence of frequent hemodialysis on the other physiological parameters and their interrelationships provides scope for further research in this area. On review of historical data, it seems that at least 50 patients in each one of the treatment and control arm will be required. However, for a study as large as this, the expertise of a statistician will be sought for power and number calculation.

## Eligibility criteria

The study will aim to include adult (>18 years of age) prevalent hemodialysis patients from at least 15 centers across the country (units that are willing to participate). Patients who are unwilling to participate or where there are pre-existing concerns about compliance will be excluded from the trial. Patients who have contraindications to a cardiac MR (metallic implants, claustrophobia) at baseline and at 1 year will be excluded. Acute renal impairment requiring dialysis is excluded. The terminally ill and patients with active infections needing long term treatment such as TB, HIV will be excluded from the trial. Patients are expected to have intact vascular access for dialysis (AVF/Tunneled catheter) at baseline. These patients will also have satisfactory clearances at baseline eKT/V >1.2 (the current recommended target- renal association guidelines).

## Data collection

Patients will be identified from multiple centers from whom, informed consent will be obtained. Those eligible to participate will be randomized into each one of the 3 arms of the trial. The randomization process will use a computer generated sequence. Patients will be enrolled into the in center daily hemodialysis program (2.5 h daily, 6 days/week). The relatively younger/and patients willing and able to train for home hemodialysis, into the nocturnal dialysis program (8 h; 6 days/week). The control group will comprise of the matched CHD patients (4 h; 3 days/week). Clearly, the nocturnal group will have a 6-8 week training period, prior to enrolment into the trial. At the start of the trial, all patients will undergo cardiac MR studies for baseline LV assessment, by the same radiographer who is blinded to the treatment arms of the patients. Before dialysis and 20 min post dialysis; BP will be recorded (9). Once a week, 20 min after hemodialysis, the same operator would measure the extracellular volume using bioimpedance analysis (10). This non-invasive tool will be used by nocturnal hemodialysis patients to record the same information. Anemia and mineral metabolism targets will be achieved according to the renal association guidance on the matter (7). At the start of the enrolment, satisfactory vascular access will be established. The nature of the vascular access and blood flows obtained, will also be recorded. Patients in all the 3 arms will be followed up to 1 year until the completion of the annual cardiovascular MR study. Laboratory data will be recorded on a 3 monthly basis. Events and changes to prescription will be recorded as they happen (Table 1).

**Table 1:** Time scale

Information to multiple centers	6 months
Assess patient eligibility	6 months
Home hemodialysis training	2 months
Enrolment into trial	1 year
Study period	1 year
Analysis and dissemination	3 months

## Data recording/ analysis

All information pertaining to the study will be stored electronically in a password protected computer system. Confidentiality will be maintained in accordance with the data protection act, 1998.

All analyses will be undertaken by the statistician who is blinded to the patient treatment arm assignment. An intention-to-treat approach will be taken for the assessment of the primary outcome. “Intention to treat” is a strategy for the analysis of RCTs that compares patients in the groups to which they were originally randomly assigned. However there is a debate about the validity of excluding specific cases within each of these categories from an intention to treat analysis (20-22). Clinical effectiveness may be overestimated if an intention to treat analysis is not done (21,22). For patients in this study who may die or receive a transplant, it will be assumed that no change in the baseline cardiac parameters have taken place. Primary study outcome (change in LVM) will be analyzed using the paired *t-*test. Statistically significant difference (p<0.05) in LVM would be 10 g (22).

## Study limitations

The study has been designed to minimize the impact of bias from influencing the results. There may be an unknown bias, from use of a convenience sample, and all efforts should be made to provide the exact demographic details, so that the outcome is disseminated to the population intended. The provision of cardiac MR study by one radiographer will ensure no inter observer bias (10). Hard end point such as mortality requires much bigger sample size and longer follows up and therefore is not feasible. The study is not designed to look at several important other secondary outcomes, as the influence of frequent hemodialysis on longevity is multifactorial. The study may give us an idea of the feasibility and adverse events rate from the technique, however, may be underpowered to give statistically significant results. Economic analysis is another aspect that is not being studied here. The effect of treatment on other secondary outcome domains will give us crucial information on the relationships between, LVMI, ECF volume and BP; on the basis of which further basic science research may become important. The study will also highlight practical issues with the provision of daily in center dialysis, with respect to trained staff and transportation of patients.

## Conclusion and future

This study was designed to look at an important aspect of hemodialysis provision, the time and frequency of it. Before embarking on a major practice change, there must be adequate evidence to support it. This study would, in the NHS setting, look at one crucial aspect, LVMI, a surrogate end point for mortality (23,24). Further psychosocial analysis of patients participating in the study are equally important to the success of such a program and should be the subject of a qualitative research in the future. The national institute of health and clinical excellence (NICE) would also have cost effectiveness of such a move, high on its priority and health economic studies on this subject are also warranted in the future.

## Authors’ contributions

CJMN and SI completed the article. FB and SAA completed the critical appraisal.

## Conflict of interests

The authors declared no competing interests.

## Ethical considerations

Ethical issues (including plagiarism, misconduct, data fabrication, falsification, double publication or submission, redundancy) have been completely observed by the authors.

## Funding/Support

None.
